# A scoring model integrating CXCL9, GDF15, FGF21, and NfL, predicts long-term mortality in type 2 diabetes: a retrospective study

**DOI:** 10.1186/s12933-025-02830-5

**Published:** 2025-07-08

**Authors:** Matilde Sbriscia, Sara Caccese, Francesca Marchegiani, Rina Recchioni, Giulia Matacchione, Chiara Giordani, Emanuele Francini, Stefano Salvioli, Maria Conte, Matteo Landolfo, Anna Rita Bonfigli, Federica Turchi, Jacopo Sabbatinelli, Fabiola Olivieri, Angelica Giuliani

**Affiliations:** 1 Clinic of Laboratory and Precision Medicine, IRCCS INRCA, Ancona, Italy; 2 Department of Medical and Surgical Sciences (DIMEC), University of Bologna, 40126 Bologna, Italy; 3 IRCCS Azienda Ospedaliero-Universitaria Di Bologna, 40138 Bologna, Italy; 4 Internal Medicine and Geriatrics, IRCCS INRCA, Ancona, Italy; 5 Scientific Direction, IRCCS INRCA, Ancona, Italy; 6 Metabolic Diseases and Diabetology, IRCCS INRCA, Ancona, Italy; 7 Department of Clinical and Molecular Sciences, Universita Politecnica Delle Marche, Via Tronto 10/A, 60126 Ancona, Italy; 8 Istituti Clinici Scientifici Maugeri IRCCS, Cardiac Rehabilitation Unit of Bari Institute, Bari, Italy

## Abstract

**Background:**

Type 2 diabetes (T2D) is a chronic metabolic disorder associated with aging, systemic inflammation, and increased long-term mortality. Identifying prognostic biomarkers may improve risk stratification and guide personalized interventions. This study aimed to evaluate the long-term prognostic value of circulating biomarkers related to inflammation, metabolic stress, and organ damage in individuals with T2D.

**Methods:**

A retrospective study was conducted on a cohort of 478 individuals with T2D, followed for a median of 16.1 years. Ten circulating biomarkers (IL-6, IL-10, CD163, CXCL9, CCL22, GDF15, IL-33, FGF21, Follistatin, and neurofilament light chain [NfL]) were quantified using an automated immunoassay platform. Kaplan–Meier survival analysis and Cox proportional hazards models were used to assess their prognostic significance for all-cause mortality. A biomarker-based scoring model was developed by integrating independent predictors of mortality. Predictive performance was evaluated in comparison with the RECODe equation, a validated risk model for diabetes complications and mortality.

**Results:**

Deceased individuals exhibited significantly higher levels of IL-6, IL-10, CXCL9, FGF21, NfL, and GDF15. Biomarker levels correlated with both microvascular and macrovascular complications, particularly neuropathy, nephropathy, retinopathy, and major adverse cardiovascular events (MACE). In multivariable Cox regression analysis, four biomarkers emerged as independent predictors of mortality: CXCL9 (HR per 1 SD increase 1.19, 95% CI 1.05–1.36, *p* = 0.006), GDF15 (HR 1.16, 95% CI 1.02–1.33, *p* = 0.032), NfL (HR 1.25, 95% CI 1.09–1.43, *p* = 0.001), and FGF21 (HR 1.20, 95% CI 1.04–1.37, *p* = 0.009). A composite biomarker score (range: 4–12) stratified individuals into distinct risk categories, with each 1-point increase in the score associated with a 55% higher mortality risk (HR 1.53, 95% CI 1.35–1.74, *p* < 0.001). The biomarker score remained independently predictive after adjusting for clinical covariates and significantly improved individual-level risk classification beyond the RECODe model, as demonstrated by net reclassification and discrimination improvement metrics.

**Conclusions:**

These findings suggest that inflammatory and metabolic stress-related biomarkers independently predict long-term mortality in T2D. The biomarker-based scoring model enhances risk stratification and improves the prognostic performance of existing clinical tools, such as the RECODe equation, potentially informing targeted clinical interventions.

**Supplementary Information:**

The online version contains supplementary material available at 10.1186/s12933-025-02830-5.

## Introduction

Type 2 diabetes mellitus (T2D) is a chronic metabolic disorder characterized by insulin resistance and impaired insulin secretion, resulting in hyperglycemia. It poses a significant global health burden, with its prevalence projected to rise to 643 million by 2030, driven by factors such as population aging, sedentary lifestyles, and increasing obesity rates [1]. T2D is associated with a markedly increased risk of all-cause mortality, primarily due to its complications, including cardiovascular disease, kidney failure, and infections. Studies indicate that individuals with T2D have a two to four times higher risk of cardiovascular-related death compared to those without diabetes [2]. Moreover, data from the Emerging Risk Factors Collaboration highlights that T2D shortens life expectancy by approximately 6 years on average. Poor glycemic control, longer disease duration, and comorbidities further exacerbate mortality risks [3]. Even in individuals with well-managed glycemia and lipid levels, a substantial residual risk of mortality remains, likely driven by chronic inflammation, metabolic stress, and progressive organ damage [4, 5]. Emphasis on early diagnosis, lifestyle modifications, and optimal medical interventions can significantly reduce the burden of diabetes-related complications and improve survival outcomes globally [6]. Identifying early markers of mortality in T2D is crucial for developing effective prevention and management strategies.

A state of low-grade, systemic, and chronic inflammation that accompanies aging, termed Inflammaging, plays a key role in influencing overall mortality risk, both in the general population [7] and among individuals with T2D [8–10]. Among circulating proinflammatory markers, C-reactive protein (CRP) and interleukin-6 (IL-6) have been extensively studied and are consistently associated with insulin resistance, endothelial dysfunction, and increased risk of cardiovascular events and mortality in T2D [8, 11, 12]. Interestingly, patients with T2D exhibit elevated IL-6 levels accompanied by a reduction in its soluble receptors sIL-6R and sgp130, which normally act as a buffer to regulate IL-6 activity [13]. This imbalance may reflect a chronic overload of the IL-6 signaling system, potentially disrupting immune homeostasis and amplifying inflammatory responses. CRP, primarily synthesized in the liver in response to IL-6, reflects systemic inflammation and vascular injury [14]. However, current prognostic tools rarely incorporate biomarkers that reflect the biological burden of inflammation, metabolic stress, and early organ damage, despite their known contribution to adverse outcomes in T2D. To date, no validated biomarker-based score exists to quantify this cumulative burden and support long-term risk stratification.

Therefore, this study aimed to evaluate the association between selected classical and novel biomarkers and long-term all-cause mortality in individuals with T2D, and to develop a composite biomarker score to improve individual risk prediction. The classical biomarkers included IL-6 and IL-10. As innovative biomarkers, we investigated the chemokines C–C motif chemokine ligand 22 (CCL22) and C-X-C motif chemokine ligand 9 (CXCL9), and CD163 as a marker of macrophage polarization. Additionally, we assessed factors associated with stress and metabolic dysfunction such as growth differentiation factor 15 (GDF15), IL-33, fibroblast growth factor 21 (FGF21), together with novel indicators of organ damage and metabolic dysfunction, including follistatin and neurofilament light chain (NfL). These markers were selected based on their known roles in inflammaging, cardiometabolic stress, and neurodegeneration in older adults and individuals with diabetes, as supported by observational evidence [9, 15–17]. In addition to assessing their individual associations with mortality, we aimed to explore their integrated prognostic potential through the development of a composite biomarker score. Translating these biomarkers’ prognostic ability into clinical practice may offer valuable insights into the impact of inflammaging and the risk of age-related comorbidities and mortality. This could have significant implications for clinical trials designed to evaluate disease-modifying therapies.

## Materials and methods

### Study population

A retrospective observational study was conducted on a previously described cohort of 568 outpatients diagnosed with type 2 diabetes mellitus (T2D) [18]. Patients were recruited at the Metabolic Diseases and Diabetology Department of IRCCS INRCA, Ancona, Italy between May 2003 and November 2006. For this investigation, a total of 478 subjects (median age: 67 years, IQR 61–72) were included, based on the availability of biological material. T2D diagnosis was determined according to the American Diabetes Association (ADA) guidelines [19], requiring at least one of the following criteria: HbA1c ≥ 6.5%, fasting blood glucose ≥ 126 mg/dL, 2-h blood glucose ≥ 200 mg/dL after an oral glucose tolerance test (OGTT), or random blood glucose ≥ 200 mg/dL in the presence of pronounced diabetes symptoms. Eligible diabetic participants met additional inclusion criteria, including a body mass index (BMI) below 40 kg/m^2^, an age range of 40 to 87 years, and the capacity and willingness to provide written informed consent. The study received approval from the Institutional Review Board of IRCCS INRCA hospital (Approval No. 34/CdB/03). Written informed consent was obtained from all participants in compliance with the principles outlined in the Declaration of Helsinki.

### Outcomes and covariates

All-cause mortality was defined as the primary endpoint and was used to derive threshold values for individual biomarkers. All-cause mortality was verified through linkage with the National Health System registry and confirmed by official death certificates. Outcome-related follow-up data were obtained from medical records, covering the period from the enrollment date (May 2003–November 2006) to the final follow-up date (December 31, 2019). Specifically, information was gathered on BMI, blood glucose control (HbA1c), renal function (estimated glomerular filtration rate [eGFR]), and the occurrence of T2D complications, assessed by diabetologists using standard diagnostic criteria, including major adverse cardiovascular events (MACE). Diabetic retinopathy was diagnosed through fundoscopy with dilated pupils and/or fluorescein angiography. Diabetic kidney disease (DKD) was characterized by a urinary albumin excretion rate exceeding 30 mg/24 h and/or eGFR < 60 mL/min/1.73 m^2^. Neuropathy was diagnosed using electromyography. MACE was defined as non-fatal myocardial infarction or cerebrovascular accident. Peripheral artery disease, including atherosclerosis obliterans and cerebrovascular conditions, was identified through patient history, physical examination, and Doppler velocimetry. The RECODe equation was computed for each participant using available clinical and laboratory data as specified in the original model, excluding the urinary albumin-to-creatinine ratio (UACR) component, which was not available at baseline and is optional according to the original publication [20]. Outcome events were assessed based on the development of T2D-related complications in those with no prior history of such conditions, all-cause mortality, and a composite endpoint of MACE and all-cause mortality.

### Biomarkers

Fasting blood samples from all participants were processed to extract serum and subsequently stored at − 80 °C. All tested analytes were analyzed using ELLA (ProteinSimple, Bio-Techne, Minneapolis, MN, USA), a platform based on a microfluidic technology that allows the performance of automated enzyme-linked immunoassays. The assay ranges of the analytes were: IL-6 (0.28–2652 pg/mL, LoD 0.11 pg/mL), IL-33 (0.75–1140 pg/mL, LoD 0.452 pg/mL), IL-10 (0.38–1446 pg/mL, LoD 0.17 pg/mL), CXCL9 (19.9–30,400 pg/mL, LoD 3.8 pg/mL), NfL (2.7–10,290 pg/mL, LoD 1.09 pg/mL), FGF21 (8.93–5450 pg/mL, LoD 3.74 pg/mL), CCL22 (0.68–2600 pg/mL, LoD 0.32 pg/mL), GDF15 (0.52–2000 pg/mL, LoD 0.21 pg/mL), Follistatin (2.77–26,410 pg/mL, LoD 0.6 pg/mL), and CD163 (19.8–12,088 pg/mL, LoD 3.23 pg/mL). All samples were run in triplicate and only samples with an intra-assay coefficient of variation below 10% were included in this study. Inter-assay coefficients of variation for the tested analytes ranged from 3 to 8%, as provided by the manufacturer and confirmed by quality control samples run across different plates.

### Statistical analysis

Continuous variables were expressed as either mean with standard deviation (SD) or median with interquartile range (IQR), depending on their distribution, which was assessed using the Shapiro–Wilk test. Biomarker concentrations below the limit of quantification (LoQ) were substituted with the LoQ divided by the square root of 2. Group comparisons of serum cardiac biomarkers were conducted using the Mann–Whitney U test and the Kruskal–Wallis test, followed by Dunn’s post-hoc analysis where applicable. Categorical variables were analyzed using the chi-square (χ^2^) test. To evaluate correlations between continuous variables, Spearman’s correlation coefficient was applied. Multivariable ANCOVA models were developed to explore factors associated with T2D complications, with biomarker concentrations as dependent variables and T2D complications as factors, adjusting for age, sex, and HbA1c. Median follow-up was estimated both conventionally and using the reverse Kaplan–Meier method to account for censoring. The relationship between biomarker levels and follow-up outcomes was examined through Kaplan–Meier survival analysis and Cox proportional hazards models, adjusted for sex, age, smoking status, hypertension, T2D duration, BMI, HbA1c, blood lipids, eGFR, and high-sensitivity C-reactive protein (hs-CRP), with 95% confidence intervals. All biomarker values were standardized as z-scores (mean = 0, SD = 1) prior to inclusion in regression models to allow comparability of effect sizes. The proportional hazards assumption for all Cox models was verified using Schoenfeld residuals, and no significant violations were detected. Logistic regression was employed to assess associations with endpoints related to MACE, given that precise event dating was not always feasible. The Evaluate Cutpoints R package was used to determine optimal biomarker thresholds for predicting mortality in T2D patients [21]. Based on these thresholds, biomarkers were classified into low, medium, and high categories. Each biomarker level was assigned a score from 1 to 3 (1 = low, 2 = medium, 3 = high), and the combined scores of three biomarkers formed a score ranging from 4 to 12. To assess discrimination performance, time-dependent AUCs at 10 years were calculated using the timeROC R package, which implements the inverse probability of censoring weighting (IPCW) method. Comparisons between AUCs from different Cox models were performed using the compare() function within the same package. To evaluate the added prognostic value of the biomarker score, the Integrated Discrimination Improvement (IDI), the continuous Net Reclassification Improvement (NRI), and the relative IDI were calculated using the survIDINRI package. The IDI.INF() function was applied to compare nested models, using linear predictors extracted from the respective Cox regressions. Confidence intervals and p-values were derived from influence function-based inference. A significant threshold of p < 0.05 was applied. All statistical analyses were performed using R (version 4.1), Jamovi (version 2.3.1), and SPSS 26.0 for Windows (SPSS Inc., Chicago, IL, USA).

## Results

### Baseline characteristics of the study population and outcomes

A total of 478 individuals with T2D were included in this study. Their baseline anthropometric, demographic, biochemical, and metabolic features according to survival state are presented in Table 1. Based on a median follow-up period of 16.1 years (IQR 13.0–16.2), we categorized the population into two groups: surviving and deceased individuals. Using the reverse Kaplan–Meier estimator, which estimates follow-up time by treating events as censored observations, the median follow-up was 11.6 years (IQR 7.1–14.1). Of the 478 subjects included in this study, 173 patients had died (36.8%). Deceased patients were older than those who survived (median age: 70 vs. 65, *p* < 0.001), and the two groups differed significantly in variables related to renal function. Additionally, the deceased group exhibited a higher rate of complications (Table 1). The overall follow-up time among participants with available biomarker values amounted to approximately 79,070 person-months (equivalent to 6,590 person-years).

**Table 1:** Baseline biochemical and demographic characteristics of individuals with type 2 diabetes

Variable	Total (T2D, N = 478)	Survived (N = 305)	Deceased (N = 173)	p-value
Age (years)	67.0 (61.0–72.0)	65 (59–69)	70 (66–74)	< 0.001
Sex (Males %)	255 (53.4%)	157 (51.5%)	98 (56.6%)	0.276
Smoking, current/former (n, %)	66 (13.8%)	44 (14.4%	22 (12.7%)	0.593
BMI (kg/m^2^)	28.0 (25.7–31.2)	28.0 (25.6–31.7)	28.0 (26.0–31.2)	0.672
Weight (kg)	77.0 (69.0–86.0)	76.5 (68.0–88.0)	77.0 (70.0–85.0)	0.966
Waist-hip ratio	0.93 (0.89–0.97)	0.93 (0.88–0.97)	0.94 (0.90–0.98)	0.018
Total cholesterol (mg/dL)	205 (180–231)	208 (181–233)	201 (179–227)	0.219
LDL-C (mg/dL)	114 (93.3–135)	114 (97.0–135)	113 (88.3–133)	0.123
HDL-C (mg/dL)	50.0 (42.0–61.0)	50.0 (43.0–62.0)	49.0 (41.0–60.0)	0.078
Triglycerides (mg/dL)	114 (83–158)	111 (79.0–155)	118 (87.0–165)	0.162
TyG Index	9.10 (8.68–9.49)	9.04 (8.63–9.47)	9.19 (8.75–9.52)	0.114
ApoA1 (mg/dL)	164 (147–186)	168 (150–189)	160 (140–182)	0.003
ApoB (mg/dL)	100 (84.0–116)	101 (85–117)	96.0 (81.8–114)	0.199
Fasting glucose (mg/dL)	152 (133–182)	150 (131–179)	156 (133–194)	0.529
Fasting insulin (μUI/mL)	5.70 (3.57–8.66)	5.80 (3.73–8.67)	5.55 (3.40–8.62)	0.796
HbA1c (%)	7.5 (6.60–8.10)	7.30 (6.60–8.10)	7.40 (6.70–8.00)	0.297
HOMA index	2.14 (1.34–3.59)	2.10 (1.39–3.51)	2.17 (1.29–3.77)	0.912
Hemoglobin (g/dL)	14.3 (13.4–15.2)	14.2 (13.5–15.2)	14.3 (13.3–15.2)	0.560
WBC (n/mm^3^)	6.62 (5.51–7.57)	6.49 (5.47–7.52)	6.78 (5.71–7.76)	0.098
Platelets (n/mm^3^)	212 (181–253)	214 (184–254)	206 (175–248)	0.466
hs-CRP (mg/L)	2.46 (1.20–4.74)	2.30 (1.16–4.42)	2.81 (1.39–6.54)	0.056
Iron (µg/dL)	80 (64.0–95.0)	80 (65–95.5)	80.0 (63.0–95.0)	0.276
Ferritin (ng/dL)	86.9 (46.7–158)	89.8 (48.6–162)	77.3 (42.6–153)	0.184
Creatinine (mg/dL)	0.90 (0.70–1.00)	0.80 (0.70–1.00)	0.90 (0.80–1.10)	< 0.001
eGFR (ml/min)	81.5 (66.8–87.0)	83.0 (70.3–93.5)	73.9 (61.5–83.9)	< 0.001
Azotemia (mg/dL)	38.0 (32.0–46.0)	37 (32–44)	42 (33–50)	< 0.001
Uric acid (mg/dL)	4.60 (4.00–5.40)	4.60 (3.90–5.30)	4.60 (4.06–5.50)	0.210
ALT (U/L)	38.5 (34.0–47.0)	40.0 (34.0–49.0)	36.0 (33.0–43.0)	0.229
AST (U/L)	19.5 (16.0–24.0)	20.0 (16.0–25.0)	19.0 (16.0–24.0)	0.002
Gamma-glutamyltransferase (U/L)	50.0 (39.0–62.0)	49.0 (40.0–62.0)	50.0 (36.8–60.0)	0.250
Total bilirubin (mg/dL)	0.6 (0.5–0.8)	0.6 (0.5–0.8)	0.6 (0.5–0.8)	0.866
Disease duration (years)	13 (7.0–21.0)	11.0 (6.0–18.0)	16 (9.25–22.0)	p < 0.001
RECODe probability of 10-year mortality (%)	13.5 (8.4–20.0)	11.0 (7.1–16.4)	18.4 (12.5–24.2)	< 0.001
Hypertension (n, %)	298 (62%)	177 (58%)	121 (70%)	0.010
MACE (n, %)	71 (14.8%)	36 (11.8%)	35 (20.2%)	0.013
Diabetic kidney disease (n, %)	61 (12.8%)	29 (9.5%)	32 (18.5%)	0.005
Neuropathy (n, %)	85 (17.8%)	43 (14.1%)	42 (24.3%)	0.005
Retinopathy (n, %)	128 (26.8%)	70 (22.9%)	58 (33.5%)	0.012
Peripheral vascular disease (n, %)	45 (9.4%)	18 (5.9%)	27 (15.6%)	< 0.001

Data are median (IQR) or number (%). *P*-values for Mann–Whitney U test (continuous variables) or χ^2^ test (categorical variables)

**Fig. 1:**
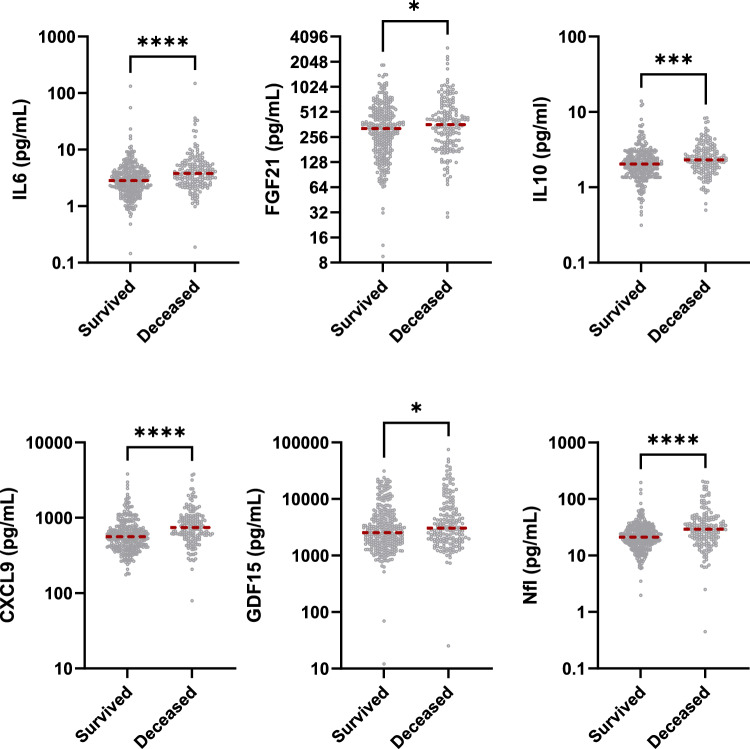
Violin plots showing statistically significant differences in biomarkers between survived and deceased subjects with T2D. Data are median (in red) and IQR. *P*-values for Mann–Whitney *U* test. *, *p* < 0.05; ****, *p* < 0.0001

Median serum concentrations of six of the assessed biomarkers were significantly higher in deceased patients (IL10, 2.04 (1.52–2.65) vs. 2.82 (1.81–3.05) pg/mL, *p* < 0.001; IL6, 2.83 (1.96–4.05) vs. 3.78 (2.54–5.46) pg/mL, *p* < 0.001; CXCL9, 561 (418–720) vs. 742 (580–1011) pg/mL, *p* < 0.001; NfL, 21.20 (15.8–29.7) vs. 29.2 (19.7–43.3) pg/mL, *p* < 0.001; GDF15 2549 (1605–5008) vs 3059 (1900–6700) pg/mL, *p* = 0.015) (Fig. 1).

The levels of the tested analytes were analyzed in relation to the presence of micro- and macrovascular complications of diabetes, including neuropathy, nephropathy, retinopathy, peripheral vascular disease (PVD), and MACE. ANCOVA analysis, adjusted for age, sex, and HbA1c, revealed a significant association between NfL and neuropathy (*p* < 0.001), nephropathy (*p* = 0.006), retinopathy (*p* < 0.001), and PVD (*p* = 0.003). GDF15 was associated with nephropathy (*p* = 0.011) and PVD (*p* = 0.035), while FGF21 showed a strong association with nephropathy (*p* < 0.001). Additionally, IL-6 was significantly linked to MACE (*p* < 0.001).

A correlation analysis was conducted to evaluate the relationships between inflammatory biomarkers and key clinical variables, including age, hs-CRP, HbA1c, and eGFR, in patients with T2D (Supplementary Table 1). CXCL9, NfL, IL-6, and GDF15 were all positively correlated with age (all *p* < 0.001), supporting their role as biomarkers of inflammaging. IL-6 showed a significant positive correlation with hs-CRP (ρ = 0.452, *p* < 0.001) and HbA1c (ρ = 0.126, *p* = 0.006), along with an inverse correlation with eGFR (ρ = − 0.173, *p* < 0.001). GDF15 was positively correlated with HbA1c (ρ = 0.181, *p* < 0.001) and hs-CRP (ρ = 0.153, *p* < 0.001) and inversely correlated with eGFR (ρ = − 0.262, *p* < 0.001). NfL and CXCL9 both showed significant inverse correlations with eGFR (ρ = − 0.332 and ρ = − 0.309, respectively; both *p* < 0.001), while CXCL9 was also positively correlated with hs-CRP (ρ = 0.139, *p* = 0.002). IL-10 was positively correlated with both HbA1c (ρ = 0.151, *p* < 0.001) and hs-CRP (ρ = 0.198, *p* < 0.001). CCL22 was inversely correlated with eGFR (ρ = − 0.160, *p* < 0.001) and positively correlated with hs-CRP (ρ = 0.105, *p* = 0.022). These findings indicate that age-related increases in NfL and GDF15, along with elevations in inflammatory cytokines, reflect the impact of inflammaging in T2D, supporting their potential as biomarkers of age-associated inflammation and metabolic decline.

### Prognostic value of NfL, GDF15, FGF21, and CXCL9 in predicting long-term mortality

The 16.1-year mortality-predictive value of the tested biomarkers, individually or in combination, was compared to a reference model incorporating key clinical and biochemical factors—sex, age, smoking status, T2D duration, BMI, HbA1c, LDL-C, eGFR, and hs-CRP. One patient was lost to follow-up; therefore, the survival analysis is based on 477 patients. Cox proportional hazards analyses, with 95% confidence intervals, for all ten variables standardized as z-scores are shown in Table 2, allowing direct comparison of effect sizes. The same analyses using biomarkers in their original continuous units are reported in Supplementary Table 2. In the multivariable analysis, CXCL9, GDF15, NfL, and FGF21 were found to be statistically significant. IL-6 and IL-10 did not retain statistical significance in the fully adjusted model. Given the upstream regulatory role of IL-6 over CRP, we repeated the analysis excluding hs-CRP; however, IL-6 remained non-significantly associated with mortality (HR 1.06, 95% CI 0.97–1.18; *p* = 0.17).

**Table 2:** Univariable and multivariable Cox regression analyses evaluating circulating biomarkers as predictors of all-cause mortality. Hazard ratios are reported per 1 standard deviation increase in biomarker level (z-score)

Predictors	Raw HR (95% CI)	Adjusted HR (95% CI)
IL-6	**1.12 (1.03–1.23)**, *p* = 0.011	1.04 (0.94–1.16)
IL-33	0.99 (0.85–1.15)	0.98 (0.83–1.15)
IL-10	**1.18 (1.05–1.32)**, *p* = 0.005	1.11 (0.99–1.26)
CXCL9 (MIG)	**1.35 (1.22–1.50)**, *p* < 0.001	**1.19 (1.05–1.36)**, *p* = 0.007
Follistatin	1.09 (0.94–1.26)	1.07 (0.93–1.24)
GDF15	**1.28 (1.14–1.44)**, *p* < 0.001	**1.16 (1.02–1.33)**, *p* = 0.032
CD163	0.94 (0.79–1.12)	0.92 (0.77–1.11)
NfL	**1.44 (1.31–1.59)**, *p* < 0.001	**1.25 (1.09–1.43)**, *p* = 0.001
CCL22	1.07 (0.92–1.23)	1.09 (0.92–1.29)
FGF21	**1.20 (1.05–1.37)**, *p* = 0.007	**1.20 (1.04–1.37)**, *p* = 0.009

Multivariable model adjusted for age, sex, HbA1c, hs-CRP, eGFR, T2D duration, hypertension and smoking status. Significant predictors are bold. The proportional hazards assumption was tested using Schoenfeld residuals; no violations were detected based on the global test, and no time-dependent effects were observed for any covariates. HR, hazard ratio

**Fig. 2:**
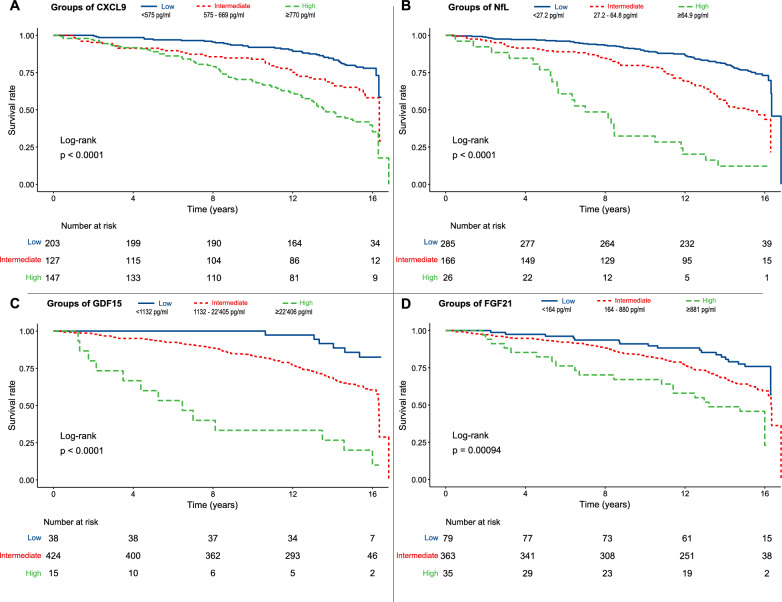
Kaplan–Meier survival estimates for **A** CXCL9, **B** NfL, **C** GDF15, **D** FGF21

Then, we categorized the four molecules that were found to be significant in the Cox analysis into three different groups (i.e., low, medium, and high). The cutoffs for T2D patients, computed to maximize the differences in survival prediction between groups, were as follows: CXCL9 low, < 517 pg/mL and high, ≥ 770 pg/mL; NfL low, < 27.2 pg/mL, high, ≥ 64.9 pg/mL; GDF15 low, < 1132 pg/mL; high, ≥ 22406 pg/mL; FGF21 low, < 164 pg/mL and high, ≥ 881 pg/mL (Fig. 2). Cox analysis confirmed that the categorized variables retained their predictive value (Supplementary Table 3).

**Fig. 3:**
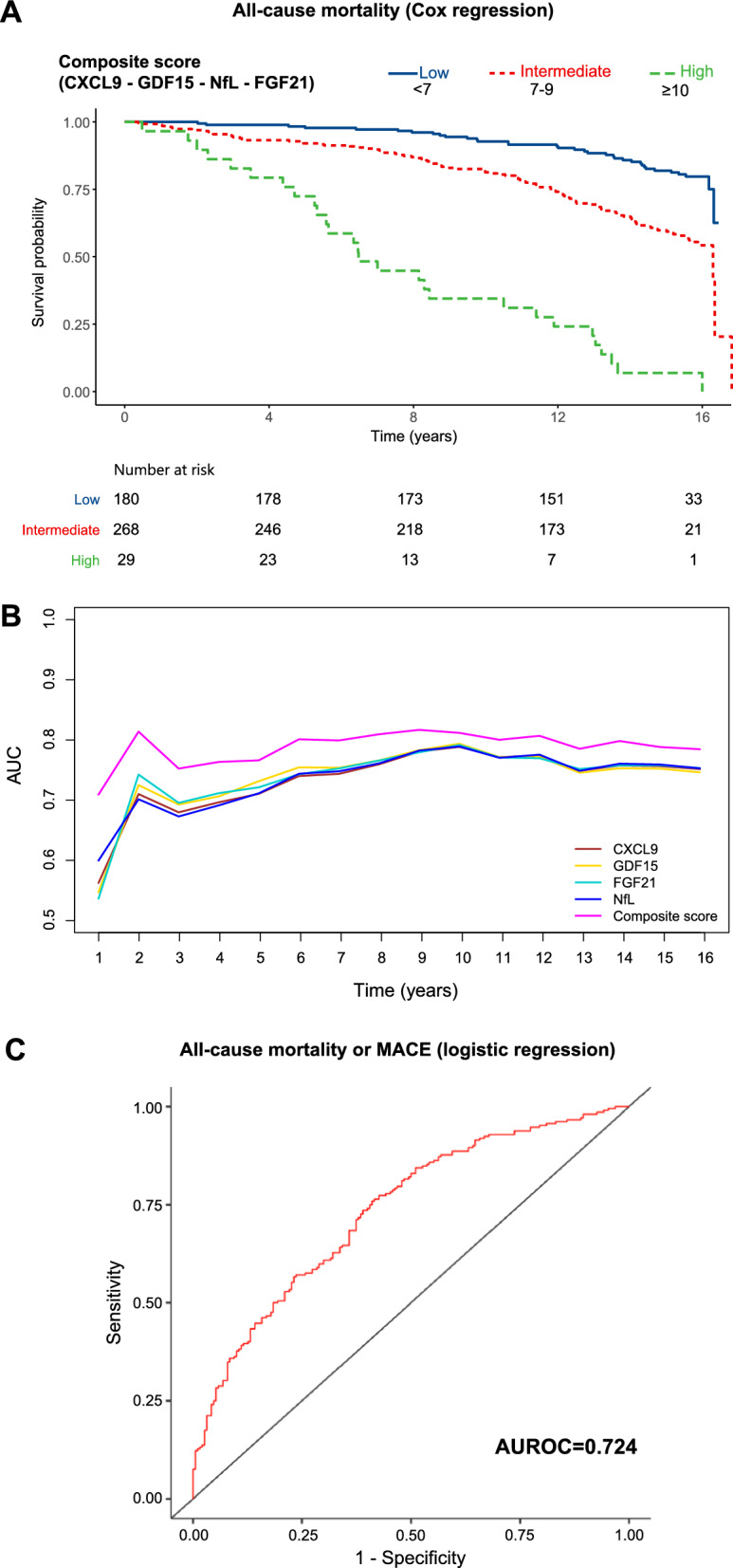
**A** Kaplan–Meier survival estimates for Composite Score derived from GDF15, CXCL9, FGF21 and NfL. **B** Time-dependent AUC curves comparing each individual biomarker (CXCL9, GDF15, FGF21, NfL) and the composite score over the follow-up period. **C** ROC curve for the logistic regression model including the biomarker-based score as predictor of the composite outcome MACE or death, evaluated without time-to-event information

To enhance the predictive accuracy of the four biomarkers, we assigned a score to each based on its level (1 = low, 2 = medium, 3 = high) using predefined cutoffs. The total biomarker score, ranging from 4 to 12, was then calculated by summing the individual scores. Higher scores were significantly associated with increased all-cause mortality (per 1-point increase: HR 1.53, 95% CI 1.35–1.74, *p* < 0.001), independent of sex, age, HbA1c, BMI, LDL, hs-CRP, hypertension and smoking status. Kaplan–Meier analysis, using the categorized risk score (low risk < 7, high risk ≥ 10), further confirmed this association (Fig. 3A).

To evaluate the temporal stability of the prognostic performance of the biomarker-based score, we generated time-dependent ROC curves comparing the composite score to each of its individual components (CXCL9, GDF15, FGF21, and NfL). As shown in Fig. 3B, the AUC of the composite score was consistently higher than those of the individual biomarkers across the 16.1-year follow-up, with the highest discriminative performance observed around year 10.

To assess whether the biomarker score added predictive value beyond established clinical models, we compared the classical RECODe equation with a biomarker-augmented model that included the total biomarker score (ranging from 4 to 12, entered as a continuous variable) for 10-year all-cause mortality prediction. The time-dependent AUC at 10 years for the RECODe model alone was 0.744 (95% CI 0.684–0.804), whereas the model combining RECODe plus the biomarker score yielded an AUC of 0.769 (95% CI 0.709–0.829). Although the absolute AUC was higher for the combined model, the difference between the two models was not statistically significant (*p* = 0.56). However, both the integrated discrimination improvement (IDI) and the continuous net reclassification improvement (NRI) analysis demonstrated significant added value of the biomarker score. The IDI was 0.097 (95% CI 0.052–0.151, *p* < 0.0001), and the continuous NRI was 0.400 (95% CI 0.269–0.494, *p* < 0.0001), indicating that the combined model more accurately assigned higher predicted risks to those who experienced events and lower risks to those who did not. Additionally, the median relative improvement of the biomarker-enhanced model was 0.075 (95% CI 0.048–0.191, *p* < 0.0001). This metric quantifies the relative gain in separation between cases and non-cases achieved by adding the biomarker score to the RECODe model. These findings suggest that, while the overall discrimination as assessed by the AUC was similar, the inclusion of the biomarker score significantly enhanced risk stratification and individual-level prediction performance over the classical RECODe equation.

The combined score was also assessed in relation to a composite endpoint of all-cause mortality or MACE among T2D patients without a prior history of MACE. Binomial logistic regression analysis, adjusted for sex, age, smoking status, hypertension, disease duration, BMI, HbA1c, LDL, eGFR, and hs-CRP, revealed that a higher score was associated with increased odds of all-cause mortality or MACE (per 1-point increase: OR 1.322, 95% CI 1.111–1.574, *p* = 0.002, Supplementary Table 4). Additionally, the ROC curve for this model demonstrated a sensitivity of 73.6% and a specificity of 61.1%, with an area under the curve (AUC) of 0.735 (Fig. 3C).

None of the individual biomarkers, or their combined score, showed a significant association with the risk of developing specific T2D complications other than MACE (data not shown).

## Discussion

Type 2 diabetes (T2D) is a chronic inflammatory condition strongly linked to aging and higher long-term mortality. In this retrospective study of individuals with T2D, we identified a composite biomarker score derived from four circulating proteins—CXCL9, NfL, GDF15, and FGF21—which reflect distinct biological pathways, including inflammation, neuronal injury, stress signaling, and metabolic dysfunction. The score was strongly and independently associated with long-term all-cause mortality and remained predictive after adjustment for established clinical risk factors. Furthermore, the score was associated with a combined endpoint of all-cause mortality and MACE.

In recent years, substantial efforts have been directed towards identifying easily accessible circulating biomarkers capable of predicting adverse outcomes, including mortality. Several novel circulating molecules have been identified, including metabolites [22], blood count parameters [23], and other inflammatory molecules [8], all of which may provide valuable prognostic insights. However, few studies have examined biomarkers predictive of long-term mortality (> 15 years) in individuals with T2D. Previous research conducted by our group has established associations between several biomarkers—including soluble ST2, cardiac troponin, advanced glycation end-products (AGEs), the receptor for AGEs (RAGE), and leukocyte DNA methylation—and an elevated risk of mortality in T2D [24–26]. These findings complement existing risk estimation models, such as ENFORCE [27] and RECODe [20], which predict 6-year and 10-year all-cause mortality, respectively, based on conventional clinical and laboratory parameters, including lipid profile, albumin-to-creatinine ratio (ACR), blood pressure, and treatment regimens.

In the present study, we observed that baseline levels of IL6, IL10, CXCL9, FGF21, NfL and GDF15 were significantly elevated in deceased patients compared to survivors. Furthermore, we demonstrated that CXCL9, NfL, FGF21, and GDF15 independently predicted all-cause mortality, even after adjustment for routine clinical predictors such as sex, age, and HbA1c. These results are consistent with growing evidence that inflammation contributes to adverse cardiometabolic outcomes independently of traditional risk factors. Recent studies have shown that immune–metabolic–vascular interactions and innate immune activation play a central role in promoting atherosclerotic burden, even in genetically predisposed individuals or those with optimal LDL-C control [28, 29]. Such findings highlight the importance of addressing residual inflammatory risk, supporting the integration of inflammatory biomarkers into risk prediction models.

Among the tested biomarkers, GDF15, a stress-induced cytokine, has been extensively investigated in age-related diseases (ARDs) and T2D due to its upregulation in response to cellular stress, aging, and metformin administration [30–32]. Targeting GDF15 has emerged as a potential therapeutic strategy for obesity and T2D. The elevated endogenous levels of GDF15 in T2D likely represent a compensatory response to restore glucose homeostasis; however, this response appears insufficient to counteract metabolic dysfunction. Only supraphysiological doses of exogenous GDF15 have demonstrated metabolic improvements in obesity-induced T2D animal models [33]. GDF15 has previously been validated as a prognostic marker of short-term mortality in T2D [34, 35], and our findings confirm its reliability as a predictor of long-term mortality.

FGF21, a stress-inducible hormone, plays a crucial role in regulating energy balance, glucose homeostasis, and lipid metabolism. FGF21 analogs have been shown to ameliorate dyslipidemia and enhance adiponectin levels in individuals with obesity and T2D, though their effects on glycemic control remain limited [36]. Previous studies have demonstrated that elevated circulating FGF21 levels predict the development of T2D [37, 38] and are associated with an increased risk of all-cause mortality in the elderly population [39] and diseasesbeyond T2D [40].

To date, limited studies have investigated circulating CXCL9 levels in individuals with diabetes [41], and none have explored its prognostic value for mortality. CXCL9 is a chemokine that plays an important role in the regulation of effector cell recruitment to sites of inflammation. The strongest evidence linking CXCL9 to ARDs has primarily focused on its role in musculoskeletal function, where it has been identified as a key predictor of increased risk for falls and fractures in older adults [42, 43], suggesting a potential association with incident frailty. Notably, a recent study found CXCL9 to be linked to cardiovascular aging in nominally healthy individuals [15]. Our findings reveal a positive correlation between CXCL9 levels and aging, underscoring the necessity of further research to clarify its role in diabetes and its broader implications in ARDs and mortality risk stratification.

NfL, a biomarker increasingly utilized in the diagnosis of neurodegenerative diseases, has also been implicated in conditions beyond diabetic neuropathy [44]. Recent research has suggested its involvement in poor glycemic control, diabetic nephropathy [45], and cognitive decline [46]. Additionally, NfL has been investigated as a potential predictor of mortality, including in geriatric patients with COVID-19 [47]. Recently, a longitudinal study based on NHANES data also reported that elevated serum NfL levels were associated with increased all-cause and cardiovascular mortality in individuals with T2D [17], in line with our findings. Both NfL and CXCL9 are associated with inflammaging; however, their roles in ARDs, T2D, and mortality risk prediction remain poorly understood, necessitating further investigation.

Since each of the investigated biomarkers [48] reflects a distinct pathophysiological pathway contributing to T2D progression and associated organ damage, we developed a composite biomarker score ranging from 4 to 12 to enhance mortality risk stratification. This score exhibited a strong independent association with all-cause mortality, even after adjusting for key clinical risk factors such as age, sex, disease duration, hypertension, smoking status, HbA1c, BMI, LDL-C, and hs-CRP. Moreover, the predictive utility of this biomarker score extended to a composite endpoint comprising all-cause mortality and MACE, highlighting its potential as a clinically relevant tool for risk assessment in T2D.

To further evaluate its added prognostic value, we compared the biomarker-based model to the RECODe equation, a validated risk tool for diabetes-related complications [20]. While the 10-year AUCs were not statistically different, the biomarker score yielded statistically significant improvements in both integrated discrimination improvement (IDI) and continuous net reclassification improvement (NRI), indicating enhanced individual-level risk classification. These findings suggest that the biomarker score provides complementary predictive information not captured by traditional clinical variables alone.

Although our study did not assess treatment effects, the biomarker score may help identify individuals at higher risk who could benefit from closer monitoring or early initiation of cardioprotective therapies. In particular, GLP-1 receptor agonists and SGLT2 inhibitors, endorsed by current guidelines for patients with elevated cardiovascular or renal risk, may be especially relevant, given their anti-inflammatory and organ-protective properties. Future studies should investigate whether biomarker-guided approaches can inform personalized therapeutic strategies.

### Strengths and limitations

This study has some limitations that should be considered. Although our findings are promising, the score was developed and tested within a single-center retrospective study, and the biomarker thresholds used to define low, medium, and high risk were derived from the same dataset. This approach may introduce overfitting and limit generalizability. To confirm the robustness and clinical utility of the score, validation in independent external cohorts, ideally in prospective settings, is essential. Such validation would help assess its reproducibility, optimize cutoffs, and support its potential integration into risk stratification frameworks in routine practice. Another limitation of our study is the absence of data on socioeconomic status and lifestyle factors such as diet and physical activity, which could have introduced residual confounding. However, key strengths include the well-characterized sample population and access to long-term mortality data. Biomarker quantification was performed using a highly sensitive and standardized multiplex immunoassay, which requires minimal serum volume while enabling precise, high-throughput measurement of multiple biomarkers. This methodology enhances sensitivity and reproducibility, making it particularly advantageous in clinical and research settings with limited sample availability. Additionally, our findings suggest potential early indicators of T2D-associated end-organ damage, which is often underdiagnosed and typically detected only at advanced stages using conventional diagnostic methods.

## Conclusions

Unlike standard diagnostic tests, which typically capture pathology only after significant tissue damage has occurred, these biomarkers may enable earlier risk assessment, allowing for timely intervention and improved disease management. While the routine clinical use of this biomarker score may currently be limited by assay availability, the growing accessibility of automated immunoassays suggests that implementation could become feasible in the near future. Further research is warranted to determine their potential clinical utility in guiding therapeutic strategies for individuals with T2D.

## Supplementary Information

Additional file1 (PDF 367 KB)

## Data Availability

The datasets generated and/or analysed during the current are available from the corresponding author on reasonable request.

## Abbreviations

ADA: American Diabetes Association
AGEs: Advanced glycation end-products
ApoA1: Apolipoprotein A1
ApoB: Apolipoprotein B
ARDs: Age-related diseases
AUC: Area under the curve
BMI: Body mass index
CCL22: C-C Motif chemokine ligand 22
CD163: Cluster of differentiation 163
CRP: C-reactive protein
CXCL9: C-X-C motif chemokine ligand 9
eGFR: Estimated glomerular filtration rate
FGF21: Fibroblast growth factor 21
GDF15: Growth Differentiation factor 15
hs-CRP: High-sensitivity C-reactive protein
HbA1c: Hemoglobin A1c
IDI: Integrated discrimination improvement
IL-6: Interleukin-6
IL-10: Interleukin-10
IL-33: Interleukin-33
LoD: Limit of detection
LoQ: Limit of quantification
MACE: Major adverse cardiovascular events
NfL: Neurofilament light chain
NRI: Net reclassification improvement
NT-proBNP: N-terminal prohormone of brain natriuretic peptide
ROC: Receiver operating characteristic
sST2: Soluble ST2
T2D: Type 2 diabetes
TyG Index: Triglyceride-glucose index
WBC: White blood cell
WHO: World Health Organization
