# Supplementary material for: A scoring model integrating CXCL9, GDF15, FGF21, and NfL, predicts long-term mortality in type 2 diabetes: a retrospective study
Source: Cardiovasc Diabetol. 2025 Jul 8;24:270. doi: 10.1186/s12933-025-02830-5 (PMC12239483; doi:10.1186/s12933-025-02830-5)

A Scoring model integrating CXCL9, GDF15, FGF21, and NfL, predicts Long-Term Mortality in Type 2 Diabetes: A Retrospective Study

Supplementary material

Supplementary Table 1. Spearman's correlation coefficients between biomarkers and selected variables.

		Age	HbA1c	eGFR	hs-CRP
**IL-10**	**Spearman's rho**	0.042	0.151***	-0.065	0.198***
	**p-value**	0.359	<.001	0.158	<.001
**IL-33**	**Spearman's rho**	0.094*	0.04	-0.002	-0.011
	**p-value**	0.04	0.385	0.96	0.817
**IL-6**	**Spearman's rho**	0.197***	0.126**	-0.173***	0.452***
	**p-value**	<.001	0.006	<.001	<.001
**CCL22**	**Spearman's rho**	0.061	0.019	-0.160***	0.105*
	**p-value**	0.183	0.685	<.001	0.022
**FST**	**Spearman's rho**	0.049	0.091*	-0.079	0.218***
	**p-value**	0.286	0.047	0.084	<.001
**CXCL9**	**Spearman's rho**	0.353***	0.003	-0.309***	0.139**
	**p-value**	<.001	0.955	<.001	0.002
**NfL**	**Spearman's rho**	0.417***	0.177***	-0.332***	-0.001
	**p-value**	<.001	<.001	<.001	0.99
**GDF-15**	**Spearman's rho**	0.180***	0.181***	-0.262***	0.153***
	**p-value**	<.001	<.001	<.001	<.001
**CD163**	**Spearman's rho**	0.022	0.185***	-0.085	0.162**
	**p-value**	0.655	<.001	0.086	0.001
**FGF-21**	**Spearman's rho**	0.021	0.056	-0.199***	0.201***
	**p-value**	0.64	0.221	<.001	<.001

* p < .05, ** p < .01, *** p < .001

Supplementary Table 2. Univariable and multivariable Cox regression analyses evaluating circulating biomarkers (as continuous variables) as predictors of all-cause mortality.

Predictors	Raw HR (95% CI)	Adjusted HR (95% CI)
IL-6 (per pg/mL)	**1.01 (1.00-1.02) (p=0.011)**	1.00 (0.99-1.02)
IL-33 (per pg/mL)	0.99 (0.89-1.10)	0.99 (0.88-1.10)
IL-10 (per pg/mL)	**1.12 (1.04-1.21) (p=0.005)**	1.08 (0.98-1.17)
CXCL9 (per 100 pg/mL)	**1.06 (1.04-1.09) (p<0.001)**	**1.04 (1.01-1.07) (p=0.007)**
Follistatin (per 100 pg/mL)	1.01 (0.99-1.03)	1.01 (0.99-1.02)
GDF15 (per 1000 pg/mL)	**1.03 (1.02-1.05) (p<0.001)**	**1.02 (1.01-1.04) (p=0.032)**
CD163 (per 10'000 pg/mL)	0.72 (0.12-4.19)	0.70 (0.11-4.40)
NfL (per 10 pg/mL)	**1.15 (1.11-1.19) (p<0.001)**	**1.08 (1.03-1.14) (p=0.002)**
CCL22 (per 100 pg/mL)	1.03 (0.96-1.11)	1.04 (0.96-1.14)
FGF21 (per 100 pg/mL)	**1.06 (1.02-1.10) (p=0.007)**	**1.05 (1.01-1.10) (p=0.009)**

Supplementary Table 3. Univariable and multivariable Cox regression analyses evaluating circulating biomarkers (as categorical variables) as predictors of all-cause mortality.

Predictors	Raw HR (95% CI)	AdjustedHR (95% CI)
	CXCL9	
Low	Ref.	Ref.
Intermediate	2.11 (1.39-3.19) p<0.001	1.66 (1.08-2.55) p=0.020
High	3.60 (2.48-5.24) p<0.001	2.50 (1.67-3.74) p<0.001
	GDF15	
Low	Ref.	Ref.
Intermediate	2.75 (1.22-6.21) p=0.015	1.41 (0.61-3.26) p=0.416
High	13.44 (5.10-35.42) p<0.001	4.90 (1.76-13.58) p<0.001
	NfL	
Low	Ref.	Ref.
Intermediate	2.18 (1.58-3.00) p<0.001	1.42 (1.01-2.00) p=0.044
High	7.77 (4.79-12.62) p<0.001	3.67 (2.05-6.58) p<0.001
	FGF21	
Low	Ref.	Ref.
Intermediate	1.82 (1.11-2.98) p=0.017	1.82 (1.10-3.03) p=0.020
High	3.31 (1.74-6.32) p<0.001	3.00 (1.52-5.90) p=0.002

Multivariable model adjusted for age, sex, HbA1c, hs-CRP, eGFR, T2D duration, hypertension and smoking status. HR, hazard ratio.

Supplementary Table 4. Logistic regression model predicting likelihood of developing the composite endpoint death or MACE in T2DM patients without previous history of MACE.

Predictors	Adjusted OR (95% CI)	p-value
Biomarker score (per 1-point increase)	1.32 (1.11-1.57)	0.002
Sex (male)	1.63 (1.03-2.56)	0.035
Age (years)	1.06 (1.03-1.10)	<0.001
BMI (kg/m^2)	1.02 (0.97-1.07)	0.550
HbA1c (%)	1.07 (0.89-1.29)	0.453
eGFR (mL/min)	0.99 (0.98-1.01)	0.404
hs-CRP (mg/L)	1.01 (0.97-1.05)	0.531
LDL-C (mg/dL)	1.00 (0.99-1.01)	0.241
Current smoker	1.34 (0.72-2.48)	0.352
Hypertension	1.84 (1.18-2.89)	0.008

Supplementary Figure 1. Violin plots showing biomarker levels in survived and deceased subjects with T2D. Data are median (in red) and IQR.

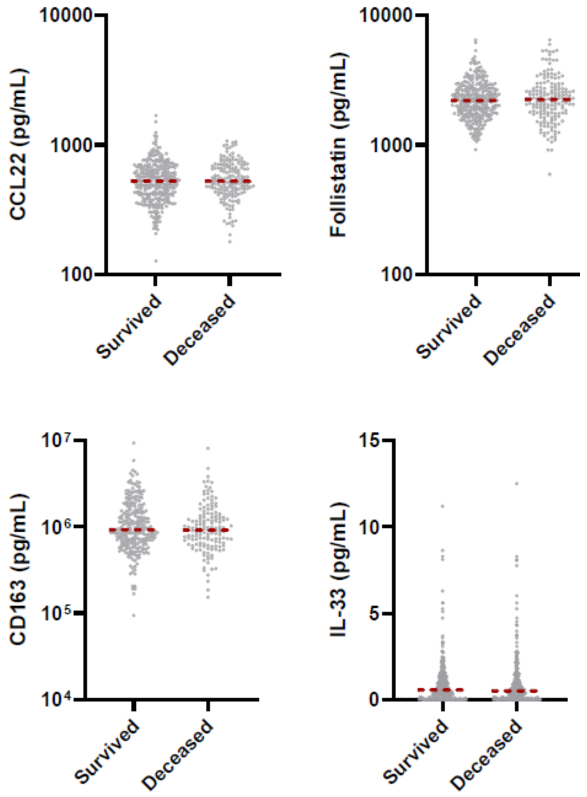

Supplementary Figure 2. Kaplan–Meier survival estimates for IL-6, IL-33, IL-10, follistatin, CCL22, and CD163.

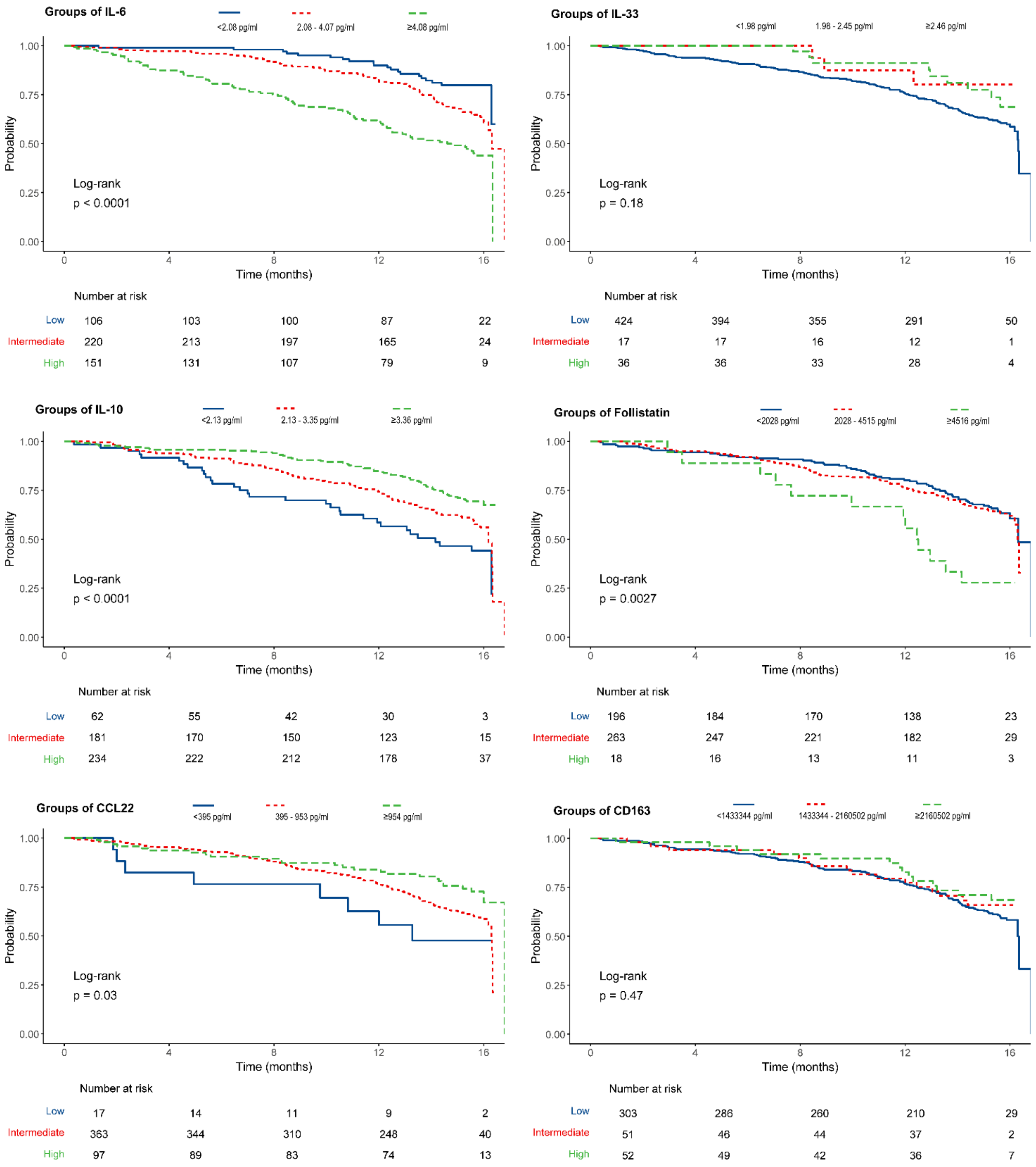

Supplement: Supplementary file 1 — Additional file1 (PDF 367 KB) [file 12933_2025_2830_MOESM1_ESM.pdf]
